# Prognostic value of histological and biological markers in pharyngeal squamous cell carcinoma: a case-control study

**DOI:** 10.1054/bjoc.1999.1188

**Published:** 2000-04-03

**Authors:** F Carinci, P F Carls

**Affiliations:** Maxillofacial Surgery, Ferrara University, Italy; Oral & Maxillofacial, John Radcliffe Hospital, Oxford, OX3 9DU, UK


sub-cloning. This arose due to a mistake in transcribing information from a larger table of MMP/TIMP oligonucleotide primer sequences when the *British Journal of Cancer* paper was being put together. However, this does not compromise the data reported in the paper, which were obtained using the correct MMP-2 primer sequences forward = 5′-GGCCCTGTCACTCCTGAGAT, reverse = 5′-GGCATCCAGGTTATCGGGGA, which as described amplify a 474 bp PCR product. The methods that we have used for MMP and TIMP quantification by RT-PCR are described in detail in Wong et al (in press).

We are grateful to Jung et al for pointing out this error, and the correct size of the MT1-MMP PCR product as 530 bp. There is certainly a need for vigilance in the use of PCR as a research tool, which we maintain in our own laboratories by subcloning and sequence analysis of RT-PCR products to confirm their identities. We agree that it is useful to identify the target gene sequences used and the positions of primer pair combinations within those sequences as a method of facilitating studies by other labs. Consequently we have drawn up this information for the MMP-2, MMP-9 and MT1-MMP targets used in our paper (Table 1). However, we caution that even with such information in hand, it is still necessary for other laboratories to confirm independently the identities of the PCR products that they obtain, using appropriate molecular criteria.

*DR Edwards*
*School of Biological Sciences, University of East Anglia, Norwich, Norfolk NR4 7TJ, UK*

#### REFERENCES

Forsyth PA, Wong H, Laing TD, Rewcastle NB, Morris DG, Muzik H, Leco KJ, Johnston RN, Brasher PMA, Sutherland G and Edwards DR (1999) Gelatinase-A (MMP-2), gelatinase-B (MMP-9) and membrane type matrix metalloproteinase-1 (MTI-MMP) are involved in different aspects of the pathophysiology of malignant gliomas. *Br J Cancer* **79**: 1828–1835

Wong H, Muzik H, Groft LL, Lafleur MA, Matouk C, Forsyth PA, Schultz GA, Wall SJ and Edwards DR (2000) Monitoring MMP and TIMP mRNA expression by RT-PCR. In: *Matrix metalloproteinase protocols*, Clark IM, (ed). Humana Press, Totoya, NJ

**Table 1** RT-PCR primer description

| Target gene | Primer sequence | Target accession number | Position | Product size (bp) | PCR cycle no. |
|---|---|---|---|---|---|
| Gelatinase-A | GGCCCTGTCACTCCTGAGAT | J03210 | 1337–1356 | 474 | 29 |
| (MMP-2) | GGCATCCAGGTTATCGGGGA | | 1810–1791 | | |
| Gelatinase-B | TGGACGATGCCTGCAACGTG | NM 004994.1 | 1554–1573 | 455 | 33 |
| (MMP-9) | GTCGTGCGTGTCCAAAGGCA | | 2008–1989 | | |
| MT1-MMP | GCCCATTGGCCAGTTCTGGCGGG | NM 004995.1 | 1178–1200 | 530 | 30 |
| (MMP-14) | CCTCGTCCACCTCAATGATGATC | | 1707–1685 | | |
| GAPDH | CGGAGTCAACGGATTTGGTCGTAT | M33197 | 78–101 | 307 | 23 |
| | AGCCTTCTCCATGGTGGTGAAGAC | | 384–361 | | |

DOI: 10.1054/ bjoc.1999.1188, available online at http://www.idealibrary.com on IDEAL®

### Prognostic value of histological and biological markers in pharyngeal squamous cell carcinoma: a case-control study

**Sir**,

We have read with great interest the article of Guerry and coworkers ‘Prognostic value of histological and biological markers in pharyngeal squamous cell carcinoma: a case-control study’ (Guerry et al, 1998). The authors present a comparison between some histological features (grading, keratinization and vascular emboli) and immunohistochemical features (expression of p53, c-*erb*-B2, Rb and bcl-2) of primary tumour biopsies in two groups of patients affected by pharyngeal cancer: patients who developed distant metastasis (DM) and patients who did not. In the case-control design each patient who developed a DM was matched to a control patient with the same tumour site, the same nodal size and level in the neck, and with an equal follow-up but free of DM. Out of 65 patients there were 45 with positive neck nodes. It was found that the risk for DM was halved in patients with tumours expressing c-*erb*-B2 compared with patients with tumours negative for c-*erb*-B2.

This result is of particular interest because it gives new perspective in the treatment planning and prognosis for these patients. However, the design of this case-control study is not completely correct.

Indeed, the two groups of patients with and without DM are only clinically homogeneous. No data regarding the immunohistochemical homogeneity of the neck nodes was presented. This is

*© 2000 Cancer Research Campaign*

essential for two reasons. Firstly, patients with positive neck nodes have a higher risk of developing DM as have those with negative neck nodes (Leemans et al, 1993; Mamelle et al, 1994). Secondly, a heterogeneity between the tumour cell populations of the primary tumour site and the neck nodes is possible. Therefore an analysis of the distribution of markers in the cervical nodes is essential. Consequently, the results are questionable at this point in time.

A case-control study in which only patients with negative neck nodes are selected will define which immunohistochemical markers are related to DM.

*F Carinci[1] and PF Carls[2]*
*[1]Assistant Professor Maxillofacial Surgery, Ferrara University, Italy; [2]Consultant Oral & Maxillofacial Surgeon, John Radcliffe Hospital, Oxford OX3 9DU, UK*

**REFERENCES**

Guerry M, Vabre L, Talbot M, et al (1998) Prognostic value of histological and biological markers in pharyngeal squamous cell carcinoma: a case control study. *Br J Cancer* **77**: 1932–1936

Leemans C, Tiwari R, Nauta J, et al (1993) Regional lymph node involvement and its significance in the development of distant metastases in head and neck carcinoma. *Cancer* **71**: 452–456

Mamelle G, Pampurik J, Luboinski B, et al (1994) Lymph node prognostic factors in head and neck squamous cell carcinomas. *Am J Surg* **168**: 494–498

DOI: 10.1054/ bjoc.1999.1189, available online at http://www.idealibrary.com on IDEAL®

### Prognostic value of histological and biological markers in pharyngeal squamous cell carcinoma – a reply

**Sir**,

As explained in our paper, the aim of our work was to study if histological or biological factors, determined on initial biopsy before treatment, could provide new prognostic information. It is a clinical question, because it can help the clinician to choose the initial treatment of the patient. For example, in the group of patients with a higher risk to develop distant metastases, the clinician could choose to begin with a general treatment (neo-adjuvant chemotherapy for example) before the loco-regional treatment. For that reason we focused on histo-biological factors which can be determined before treatment.

The idea of studying immunohistochemical markers on neck nodes is interesting, but it is a different question: it can only be determined after surgery.

Nevertheless, the suggestion of Drs Carinci and Carls is important. We are currently performing new studies so as to better understand our results. We have seen that the expression of c-*erb*-B2 can be present in non-transformed mucosa and disappear in adjacent transformed mucosa (discussion of our paper). It will be interesting to see if, when expressed in tumour, it can also disappear in involved lymph nodes.

Thank you for your interest in our work.

*M Guerry, L Vabre, M Talbot, G Mamelle, AM Leridant, C Hill, J Bosq, B Luboinski and F Janot*
*Department de Chirugie Cervico-faciale, Institut Gustave Roussy, 39 Rue Camille, Desmoulins 94805, France*

*© 2000 Cancer Research Campaign*

